# mRNA Vaccines Targeting the Conserved SARS-CoV-2 Fusion Machinery Elicit Cross-Variant and Robust Immunity

**DOI:** 10.3390/vaccines14090831

**Published:** 2026-09-21

**Authors:** Jun Li, Ke-Meng Li, Shu-Heng Yu, Ming-Hua Li, Xiao-Li Feng, Wei Pang, Yong-Tang Zheng, Jian Zhang

**Affiliations:** 1 Center for Life Sciences, School of Life Sciences, Yunnan University, Kunming 650091, China; lijun@stu.ynu.edu.cn (J.L.); 32620250156556@stu.xmu.edu.cn (K.-M.L.); 2 Southwestern United Graduate School, Kunming 650500, China; 3 State Key Laboratory of Vaccines for Infectious Diseases, Xiang An Biomedicine Laboratory, Department of Laboratory Medicine, School of Public Health, School of Life Sciences, Xiamen University, Xiamen 361102, China; 4 State Key Laboratory of Genetic Evolution & Animal Models, Key Laboratory of Bioactive Peptides of Yunnan Province, KIZ-CUHK Joint Laboratory of Bioresources and Molecular Research in Common Diseases, Kunming Institute of Zoology, Chinese Academy of Sciences, Kunming 650223, China; yushuheng22@mail.ucas.ac.cn (S.-H.Y.); zhengyt@mail.kiz.ac.cn (Y.-T.Z.); 5 Kunming National High-Level Biosafety Research Center for Non-Human Primates, Center for Biosafety Mega-Science, Kunming Institute of Zoology, Chinese Academy of Sciences, Kunming 650107, China; limh@mail.kiz.ac.cn (M.-H.L.); fengxiaoli1@mail.kiz.ac.cn (X.-L.F.); 6 Yunnan Provincial Key Laboratory of Cross-Border Infectious Diseases Control & Drug Innovation, Department of Pathogen Biology and Immunology, Faculty of Basic Medical Science, Kunming Medical University, Kunming 650118, China; 7 School of Life Sciences and Obstetrics, Gynecology Hospital, Fudan University, Shanghai 200433, China

**Keywords:** SARS-CoV-2, mRNA vaccine, heptad repeat (HR), cross-variant neutralizing antibody, cellular immunity

## Abstract

Background/Objectives: Despite the gradual waning of global interest in SARS-CoV-2, the continued evolution and emergence of viral variants underscore the need for broadly protective vaccines. Vaccines targeting the highly mutable S1 subunit of the spike protein have shown limited breadth of protection, whereas conserved regions within the S2 fusion machinery remain critical targets for the development of vaccines effective against future variants. This study evaluated two mRNA-lipid nanoparticle (mRNA-LNP) vaccines encoding HR121, a conserved HR1-HR2-HR1 fusion-intermediate immunogen, in comparison with a recombinant HR121 protein vaccine. Methods: Two mRNA vaccine candidates, TM-HR121 and Fc-HR121, were designed to present HR121 through distinct antigen presentation strategies by incorporating a transmembrane anchor or an IgG Fc domain, respectively. Immunogenicity and protective efficacy were assessed in New Zealand White rabbits, BALB/c mice, and Golden Syrian hamsters. Results: Both mRNA vaccines elicited robust HR121-specific binding antibody responses and broad neutralizing activity against seven SARS-CoV-2 pseudoviruses in rabbits. In mice, the mRNA vaccines induced higher peak IgG titers, a more balanced IgG1/IgG2a profile, stronger antigen-specific IFN-γ responses, and enhanced T-cell responses compared with the recombinant protein vaccine, while maintaining detectable IL-4 responses. In hamsters, immunization with the HR121 mRNA vaccines significantly reduced lung viral RNA levels and attenuated pulmonary pathology and nucleocapsid antigen expression following authentic SARS-CoV-2 challenge. Conclusions: HR121-based mRNA-LNP vaccines successfully elicited cross-variant humoral and cellular immune responses and conferred protective efficacy in small-animal models. These findings support HR121 mRNA-LNP vaccines as a promising strategy for the development of broadly protective coronavirus vaccines targeting conserved S2 fusion epitopes.

## 1. Introduction

The SARS-CoV-2 pandemic and the continuous emergence of novel variants present enduring challenges to global public health [1,2,3]. Since its inception, multiple variants of concern (VoCs), such as Delta and Omicron, have emerged. A hallmark of these variants is the accumulation of diverse mutations, most of which are located within the receptor-binding domain (RBD) and N-terminal domain (NTD) of the immunodominant S1 subunit of the spike (S) protein [4,5]. The efficacy of monoclonal antibodies developed against the original strains and of early vaccines mainly based on the S1 subunit to block ACE2 interactions was greatly diminished [6,7]. This antigen shift underscores the urgent requirement for vaccines capable of providing “variant-proof” protection by targeting more conserved, mutation-resistant regions of the virus [8].

Unlike the highly variable S1 subunit, the S2 subunit—which mediates the essential membrane fusion process—demonstrates high sequence conservation both across SARS-CoV-2 variants and within betacoronaviruses [9]. During the membrane fusion process between viruses and host cells, the S1 subunit dissociates after receptor binding, triggering a series of irreversible conformational rearrangements in S2. A pivotal step in this process is the refolding of the heptad repeat 1 (HR1) and heptad repeat 2 (HR2) domains into a stable post-fusion six-helix bundle (6-HB). This structure acts as a molecular zipper, pulling the viral and cell membranes into tight proximity to promote membrane fusion [10]. The functional indispensability of this step imposes stringent evolutionary constraints; mutations occurring within HR1 or HR2 usually carry substantial fitness costs, which ensures that these regions remain highly conserved across distantly related viral lineages. Such a high degree of conservation makes them highly appealing targets for broad-spectrum antiviral strategies, including fusion-inhibitory peptides and vaccines engineered to induce antibodies targeting epitopes located within these regions [11,12].

In our previous study, we developed the recombinant protein vaccines HR121 and HR212 based on the HR1 and HR2 regions, respectively. Both vaccine candidates could induce cross-reactive neutralizing antibodies, presumably by sterically hindering the formation of the functional 6-HB [13,14].

The clinical success of mRNA-LNP (lipid nanoparticle) SARS-CoV-2 vaccines offers a compelling solution to these challenges. By enabling direct intracellular antigen expression, mRNA vaccines facilitate efficient antigen presentation through both the MHC I and MHC II pathways. Consequently, they can activate both potent CD8^+^ T-cell and Th1-biased CD4^+^ T-cell responses while inducing robust humoral immunity [15,16]. This unique capacity to mobilize multiple arms of the adaptive immune system makes the mRNA platform ideally suited for vaccines targeting conserved regions. We hypothesized that mRNA-LNP delivery of HR121 would induce broader neutralizing antibody responses and stronger, more balanced cellular immunity than prokaryotically expressed recombinant HR121, thereby improving protection against authentic SARS-CoV-2 challenge.

We previously designed the recombinant protein vaccine HR121, which incorporates the HR1 and HR2 domains derived from the prototype SARS-CoV-2 strain [13]. The HR121 construct, structured as HR1-HR2-HR1, stabilizes and presents the functional fusion intermediate conformation and can elicit strong humoral immunity against multiple SARS-CoV-2 variants of concern (VoCs). Building on these findings, the present study developed two distinct SARS-CoV-2 mRNA vaccine constructs: TM-HR121, which integrates a transmembrane domain (TM) to anchor HR121 on the cell surface and mimic the native membrane-bound topology of the S2 fusion machinery, and Fc-HR121, which incorporates an Fc domain to promote dimerization, enhance stability, facilitate Fc receptor-mediated uptake [17,18] and maximize the exposure of conserved S2 neutralizing epitopes. These two formats were designed as complementary antigen presentation strategies: one displaying the immunogen in a membrane-proximal conformation to preserve conformational epitopes [17,18] and the other providing an Fc-fused immunogen with enhanced stability and prolonged bioavailability [19,20]. We demonstrated that the two mRNA vaccine candidates induced significantly stronger cellular immunity than their recombinant protein counterpart and offered better protection against live-virus challenge in Golden Syrian hamsters. This work thus provides a proof-of-concept for developing HR121-based cross-variant mRNA vaccines against SARS-CoV-2.

## 2. Materials and Methods

### 2.1. Cells, Viruses, and Animals

Authentic SARS-CoV-2 strains were provided by the Kunming Institute of Zoology (KIZ), Chinese Academy of Sciences (CAS). All authentic virus amplification and in vivo challenge experiments were strictly conducted in the Animal Biosafety Level 3 (BSL-3) facility at KIZ. The VSV-G pseudotyped virus backbone (G*ΔG-VSV-Rluc) and the S protein packaging plasmids were constructed as previously described [13]. HEK-293T cells stably expressing human ACE2 (293T-ACE2) were generously provided by Prof. You-Chun Wang (Institute of Medical Biology, Chinese Academy of Medical Sciences). All cell lines were cultured in Dulbecco’s Modified Eagle Medium (DMEM) supplemented with 10% fetal bovine serum (FBS; Gibco, Grand Island, NY, USA) at 37 °C with 5% CO_2_. Female BALB/c mice and New Zealand White rabbits were supplied by the Animal Center of Yunnan University. Male Golden Syrian hamsters were purchased from Vital River Laboratory Animal Technology Co., Ltd. (Beijing, China).

### 2.2. Plasmid Construction, Protein Purification, and Structural Modeling

The DNA sequence encoding the HR121 antigen was synthesized (Sangon Biotech, Shanghai, China) and subcloned into the EcoRI and XhoI restriction sites of the pET-30a prokaryotic expression vector. The HR121 antigen was designed to contain the HR1, HR2, and HR1 domains connected in tandem by flexible glycine–serine linkers. Specifically, in the TM-HR121 construct, the HR1 and HR2 domains were joined by a GGSGG linker, and the HR2 and the second HR1 domain were joined by an SGGRGG linker. In the Fc-HR121 construct, an additional GGGGS linker was introduced between the IgG Fc domain and the N-terminal HR1 domain. The complete amino acid sequences of all constructs are provided in Table S1. The recombinant HR121 protein was expressed in Escherichia coli BL21 (DE3) competent cells. Briefly, protein expression was induced by the addition of 0.5 mM isopropyl β-D-1-thiogalactopyranoside (IPTG) at 16 °C for 16 h. Bacterial pellets were harvested via centrifugation, resuspended in lysis buffer, and disrupted by sonication. Following centrifugation to remove cellular debris, the target protein expressed from pET-30a was purified using a Ni-Sepharose 6 FF affinity column (GE Healthcare, Shanghai, China). The three-dimensional structure of HR121 was predicted using AlphaFold 3, and structural visualizations were rendered using PyMOL (version 2.5.4).

### 2.3. Immunofluorescence Analysis of mRNA Expression and Localization

293T cells were seeded onto sterile glass coverslips placed in 24-well plates at a density of 2 × 10^5^ cells per well in 500 μL of culture medium and cultured for 12–16 h. When the cells reached 70–80% confluence, they were transfected with either the in vitro-transcribed mRNA constructs (TM-HR121 or Fc-HR121) or a plasmid DNA encoding the HR121 antigen as an intracellular expression control, using Lipofectamine™ 2000 transfection reagent (Invitrogen, Carlsbad, CA, USA) according to the manufacturer’s instructions. At 48 h post-transfection, cells were washed once with PBS and fixed with 500 μL of 4% paraformaldehyde in PBS for 10 min at room temperature. After removing the fixative and washing once with PBS, cells were permeabilized with 0.1% Triton X-100 (Sigma, St. Louis, MO, USA) in PBS for 15 min at room temperature. Following an additional PBS wash, cells were blocked with 500 μL of 1% bovine serum albumin (BSA; Invitrogen, Carlsbad, CA, USA) in PBS for 30 min at room temperature. Cells were then incubated with HR121-specific rabbit immune serum (1:100 dilution in blocking buffer) for 1 h at room temperature. After three washes with PBS, cells were incubated with Alexa Fluor™ 647-conjugated goat anti-rabbit IgG (H + L) highly cross-adsorbed secondary antibody (Invitrogen, 1:1000 dilution) for 1 h at room temperature in the dark. Following three additional washes with PBS, coverslips were mounted onto glass slides using a fluorescence mounting medium containing DAPI and visualized under a fluorescence microscope.

### 2.4. mRNA Vaccine Preparation and LNP Encapsulation

The mRNA constructs were synthesized in vitro via T7 RNA polymerase-mediated transcription. The codon-optimized open reading frames (ORFs) for the specific antigens, flanked by optimized 5′ and 3′ untranslated regions (UTRs), were synthesized by GenScript (Shanghai, China) and cloned downstream of a T7 promoter. During in vitro transcription, the mRNAs encoding TM-HR121 and Fc-HR121 were synthesized using N1-methylpseudouridine-5′-triphosphate (m1ΨTP) in place of uridine-5′-triphosphate (UTP), while ATP, CTP, and GTP were used in their unmodified forms. Following sequence design and synthesis, the formulation and lipid nanoparticle (LNP) encapsulation of the mRNA vaccines (TM-HR121 and Fc-HR121) were outsourced to RNACure Biopharma (Shanghai, China).

### 2.5. Animal Immunization and Serum Collection

Immunizations were conducted using a prime-boost regimen. Rabbit Model: Four adult female New Zealand White rabbits (average weight: 2.5 kg) were randomly allocated to receive either the TM-HR121 or the Fc-HR121 mRNA vaccine. Animals were intramuscularly (i.m.) immunized with 50 μg of mRNA per dose, with a booster administered at a 21-day interval. Blood was collected from the marginal ear vein, and pre-immune sera were retained as negative controls. Mouse Model: Female BALB/c mice (6–8 weeks old) were divided into respective cohorts (*n* = 6 per group). Mice were immunized i.m. with either mRNA candidates (2 μg or 5 μg) or the recombinant HR121 protein formulated with 2% Alhydrogel adjuvant (InvivoGen, San Diego, CA, USA). A mock group received 50 μL of PBS. Immunizations were spaced 21 days apart. Sera were collected biweekly. At day 56 post-initial immunization, mice were euthanized for splenocyte isolation. Hamster Model: Male Golden Syrian hamsters (8 weeks old) were designated for the authentic virus challenge study. Animals (n = 8 per group) were immunized with the mRNA candidates or normal saline (NS, serving as the mock control) every 21 days. Sera were collected prior to the viral challenge to confirm antibody titers.

Female New Zealand White rabbits and female BALB/c mice were used for immunization studies because females generally produce stronger and more consistent humoral immune responses [21], whereas male Golden Syrian hamsters were used for viral challenge because male hamsters exhibit more consistent and severe disease manifestations [22], thereby improving the sensitivity of the protection assessment.

The animal study was approved by the Laboratory Animal Welfare and Ethics Committee of Yunnan University (approval number: YNU20241061). All animal experiments were carried out at the Laboratory Animal Center of Yunnan University and were performed in accordance with relevant institutional guidelines and regulations.

### 2.6. ELISA for Immunogenicity Assessment

An indirect enzyme-linked immunosorbent assay (ELISA) was employed to quantify HR121-specific binding antibodies. Briefly, 96-well microtiter plates were coated overnight at 4 °C with 100 μL of purified HR121 recombinant protein (1 μg/mL) diluted in carbonate-bicarbonate coating buffer (15 mM Na_2_CO_3_, 35 mM NaHCO_3_). Plates were blocked with 1% bovine serum albumin (BSA) in PBS containing 0.05% Tween 20 (PBST) for 1 h at room temperature. Mouse sera were initially diluted 1:100, followed by a 3- to 5-fold serial dilution in PBS (comprising 11 gradient points), and incubated in the coated plates for 1 h at 37 °C. Following rigorous PBST washes, the plates were incubated for 1 h at 37 °C with the appropriate horseradish peroxidase (HRP)-conjugated secondary antibody, including goat anti-mouse IgG (HRP) (ZSGB-Bio, Beijing, China, Cat# ZB-2305), goat anti-rabbit IgG (HRP) (Abcam, Cambridge, UK, Cat# ab205718), or rabbit anti-Armenian hamster IgG H&L (HRP) (Abcam, Cat# ab5745), depending on the primary antibody used. The reaction was developed using 3,3′,5,5′-tetramethylbenzidine (TMB) substrate (Solarbio, Beijing, China, Cat# PR1210) and quenched with 1 M H_2_SO_4_ after approximately 10 min. The optical density (OD) was measured at 450 nm using a Synergy H1 microplate reader (BioTek, Winooski, VT, USA). The cutoff value for determining the endpoint titer was established as the mean OD of the negative control wells plus 0.12.

### 2.7. Pseudovirus Production and Neutralization Assay

Pseudotyped virus neutralization assays were performed as previously described [13]. Briefly, HEK-293T cells were transfected with plasmids encoding specific SARS-CoV-2 Spike variants. After 24 h, the cells were infected with the G*ΔG-VSV-Rluc backbone at a multiplicity of infection (M.O.I.) of 1. Supernatants containing the pseudoviruses were harvested at 24 h and 48 h post-infection, centrifuged to remove cellular debris, aliquoted, and titrated. A panel of seven VSV-based pseudoviruses was generated (listed in Table S2), encompassing the ancestral strain (WT) and variants including D614G, XBB.1.16, XBB.1.16.1, XBB.2.3, BA.2.86, and JN.1. For the neutralization assay, 293T-ACE2 cells were seeded into 96-well plates (1 × 10^4^ cells/well). The following day, rabbit sera (starting at a 1:20 dilution) were serially diluted 3-fold. The diluted sera (60 μL) were mixed with an equal volume of pseudovirus (M.O.I. = 0.1) and incubated at 37 °C for 1 h. The virus–serum mixtures (100 μL) were then transferred onto the pre-seeded 293T-ACE2 cells and cultured for 24 h. Intracellular Renilla luciferase activity was quantified using a commercial assay kit (Promega, Madison, WI, USA) and read on a microplate luminescence reader [23].

### 2.8. Authentic Virus Challenge and Viral Load Measurement

Hamsters were anesthetized with isoflurane and challenged intranasally with authentic SARS-CoV-2 (WT, XBB, and JN.1.5) at a dose of 1 × 10^4^ TCID_50_ in a total volume of 100 μL per animal following immunization. At 4 days post-infection (dpi), the hamsters were humanely euthanized and necropsied. Lung tissues were collected, and total RNA was extracted using TRIzol reagent (Takara, Tokyo, Japan), with RNA concentrations determined using a NanoDrop 2000 spectrophotometer (Thermo Fisher Scientific, Waltham, MA, USA). Viral RNA from culture supernatants was extracted using a Roche RNA isolation kit (Basel, Switzerland). Viral loads were quantified using a BIO-RAD CFX96 Real-Time PCR Detection System. Primers specific for the genomic RNA (gRNA) and subgenomic RNA (sgRNA) of SARS-CoV-2 were utilized as described in previous protocols [13]. All animal procedures were reviewed and approved by the Animal Ethics Committee of the Kunming Institute of Zoology, Chinese Academy of Sciences (Approval No. SMKX-2021-01-006), and were performed in accordance with the institutional guidelines for the care and use of laboratory animals.

### 2.9. Histological and Immunohistochemical Analysis

Lung tissues from infected animals were harvested, fixed in 4% paraformaldehyde for 3 days, embedded in paraffin, and sectioned at a thickness of 3 μm. To assess pulmonary pathology, sections were subjected to standard Hematoxylin and Eosin (H&E) staining and analyzed by Servicebio (Wuhan, China). Parallel sections were utilized for immunohistochemistry (IHC) and stained with an antibody against the SARS-CoV-2 nucleocapsid (N) protein (Sino Biological, Beijing, China) to visually evaluate the spatial distribution and replication levels of the viral antigen within the lung parenchyma.

### 2.10. Isolation of Splenocytes and Evaluation of Cellular Immunity

Spleens from immunized mice were harvested, mechanically disrupted, and passed through a 70-μm cell strainer to obtain a single-cell suspension. Mononuclear cells were carefully isolated using Ficoll-Paque PREMIUM (Cytiva, Medford, OR, USA) density gradient centrifugation and subsequently counted. Flow Cytometry (FCM): To evaluate T-cell subsets, approximately 1 × 10^6^ cells per tube were initially incubated with 0.25 μg of TruStain FcX™ PLUS (anti-mouse CD16/32, BioLegend, Beijing, China) antibody on ice for 10 min to block Fc receptors. For surface staining, cells were incubated with 5 μL APC anti-mouse CD3e (BioLegend), 2 μL FITC anti-mouse CD4 (BioLegend), and 5 μL PE anti-mouse CD8a (BioLegend) in the dark at 4 °C for 30 min. After staining, cells were washed with 1 mL of Cell Staining Buffer, centrifuged at 350× *g* for 5 min, resuspended in 100 μL of buffer, and acquired on a flow cytometer (Agilent NovoCyte, Santa Clara, CA, USA) for analysis. ELISpot Assay: Antigen-specific T-cell function was evaluated using Mouse IFN-γ and IL-4 ELISpot PLUS (ALP) kits (Mabtech, Shenzheng, China) following the manufacturer’s instructions. Splenocytes (4 × 10^5^ cells/well in 80 μL) were seeded into the plates and stimulated with a customized HR121 peptide pool (final concentration: 5 μg/mL per peptide). Plates were incubated at 37 °C for 24 h. Spot-forming units (SFUs) were captured using a Mabtech IRIS™ 2 reader (Mabtech, Nacka, Sweden) and quantified via automated software counting.

### 2.11. Statistical Analysis

All statistical analyses were executed using GraphPad Prism software (version 8.0.1). *p* values are indicated in the respective figure legends. Binding antibody (bAb) and neutralizing antibody (nAb) titers were expressed as geometric means ± geometric standard deviation (SD). Viral gRNA and sgRNA copy numbers were presented as medians ± interquartile ranges (IQRs). Statistical significance between the HR121-vaccinated groups and control cohorts was determined using a two-tailed Mann–Whitney U test or Wilcoxon signed-rank test.

## 3. Results

### 3.1. Design, Characterization, and Expression of HR121-Based Vaccine Candidates

**Figure 1:**
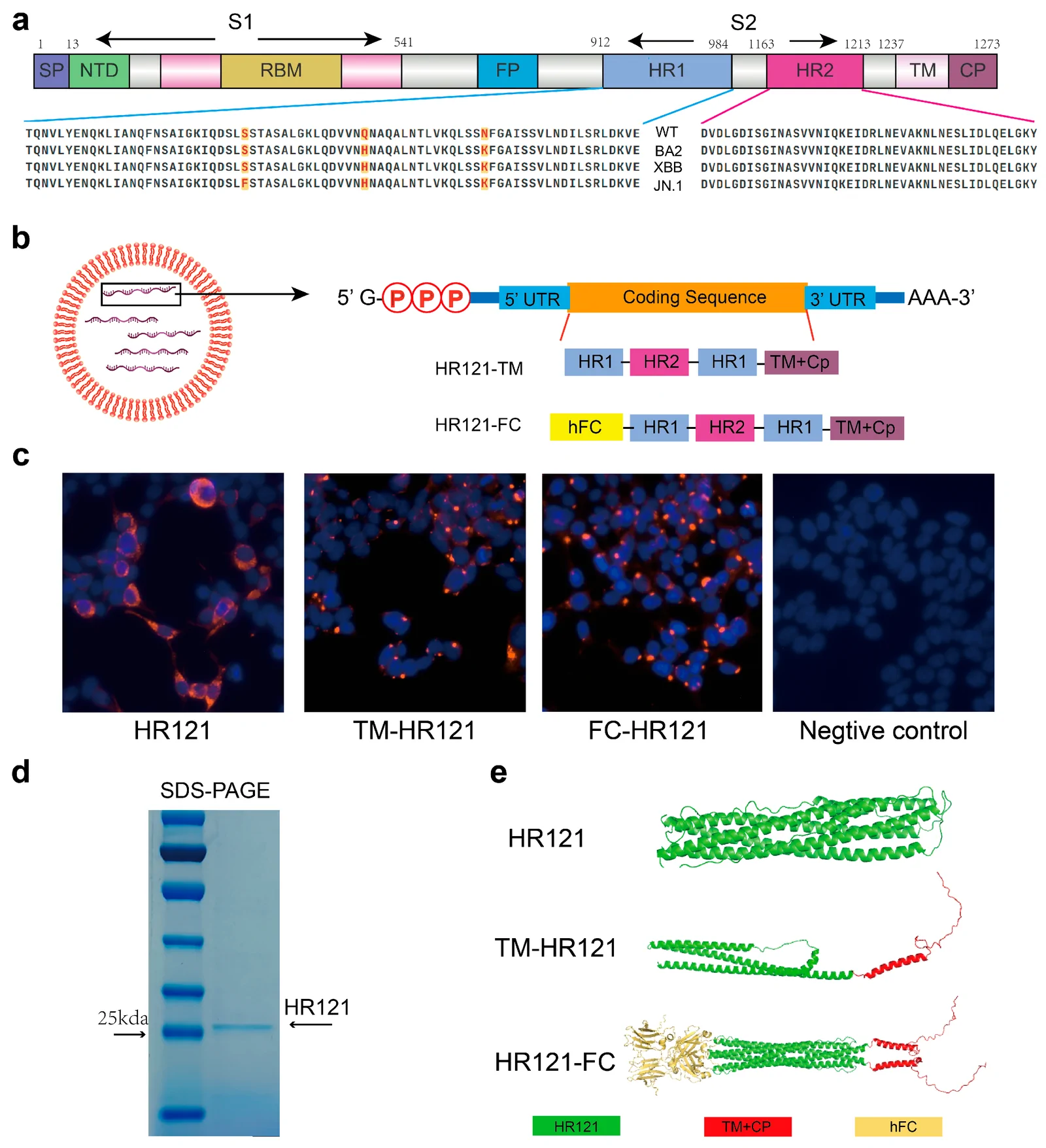
Design, structural modeling, and in vitro expression of HR121 vaccine candidates. (**a**) Schematic representation of the SARS-CoV-2 spike protein S2 subunit, highlighting the highly conserved heptad repeat 1 (HR1) and heptad repeat 2 (HR2) domains. (**b**) Illustration of the three vaccine platforms: TM-HR121 (transmembrane-anchored mRNA), Fc-HR121 (Fc-fused mRNA), and recombinant HR121 protein. (**c**) Representative immunofluorescence images of TM-HR121 and Fc-HR121 expression in transfected cells. (**d**) SDS-PAGE analysis showing the purity and molecular weight of the prokaryotically expressed recombinant HR121 protein. (**e**) Three-dimensional structural prediction of the HR121 antigen generated by AlphaFold 3 and visualized in PyMOL.

We previously developed the cross-variant recombinant protein vaccine HR121 against SARS-CoV-2 and its variants by linking the highly conserved HR1 and HR2 domains from the prototype SARS-CoV-2 strain in a sequential HR1-HR2-HR1 arrangement (Figure 1a) [13]. Here, we incorporated two naturally occurring mutations among the SARS-CoV-2 emerging variants, Q954H and N969K, into the HR1 domains of HR121 [24]. The Q954H mutation has been shown to enhance the stability of the postfusion HR1–HR2 core, thereby promoting membrane fusion. In addition, Q954H has been associated with reduced neutralization efficacy of certain fusion inhibitors against Omicron [25]. The N969K mutation induces a marked conformational displacement of the HR2 backbone in the HR1–HR2 postfusion bundle, altering the fusion machinery and reducing susceptibility to prototype-based fusion inhibitors [26]. These functional changes are linked to the increased fusogenicity observed in emerging variants. To convert the established HR121 immunogen into mRNA vaccine formats, we designed a transmembrane-anchored HR121 (TM-HR121) and a human IgG Fc-fused HR121 (Fc-HR121) mRNA-LNP vaccine candidate, as illustrated in Figure 1b. The full amino acid sequences and domain organizations of both constructs are provided in Table S1. To verify that the designed mRNA constructs could be efficiently translated and correctly localized, 293T cells were transiently transfected with TM-HR121 or Fc-HR121 mRNA, or with a plasmid DNA encoding HR121 as an intracellular expression control, and analyzed by immunofluorescence staining. Cells transfected with either mRNA construct showed strong membrane-associated fluorescence, consistent with cell surface display of TM-HR121 and extracellular expression of Fc-HR121, whereas cells transfected with the DNA plasmid exhibited predominantly intracellular staining (Figure 1c). This difference confirmed that the mRNA vaccines directed expression to the cell surface or extracellular compartment as intended [13] (Figure 1d). Using AlphaFold3 to visualize the predicted domain organization, it was shown that TM-HR121 is similar to HR121 and Fc-HR121 is a dimer of HR121 (Figure 1e).

### 3.2. Both HR121 mRNA Vaccines Elicited High Levels of Broadly Neutralizing Antibodies Against Multiple SARS-CoV-2 Variants in Rabbits

**Figure 2:**
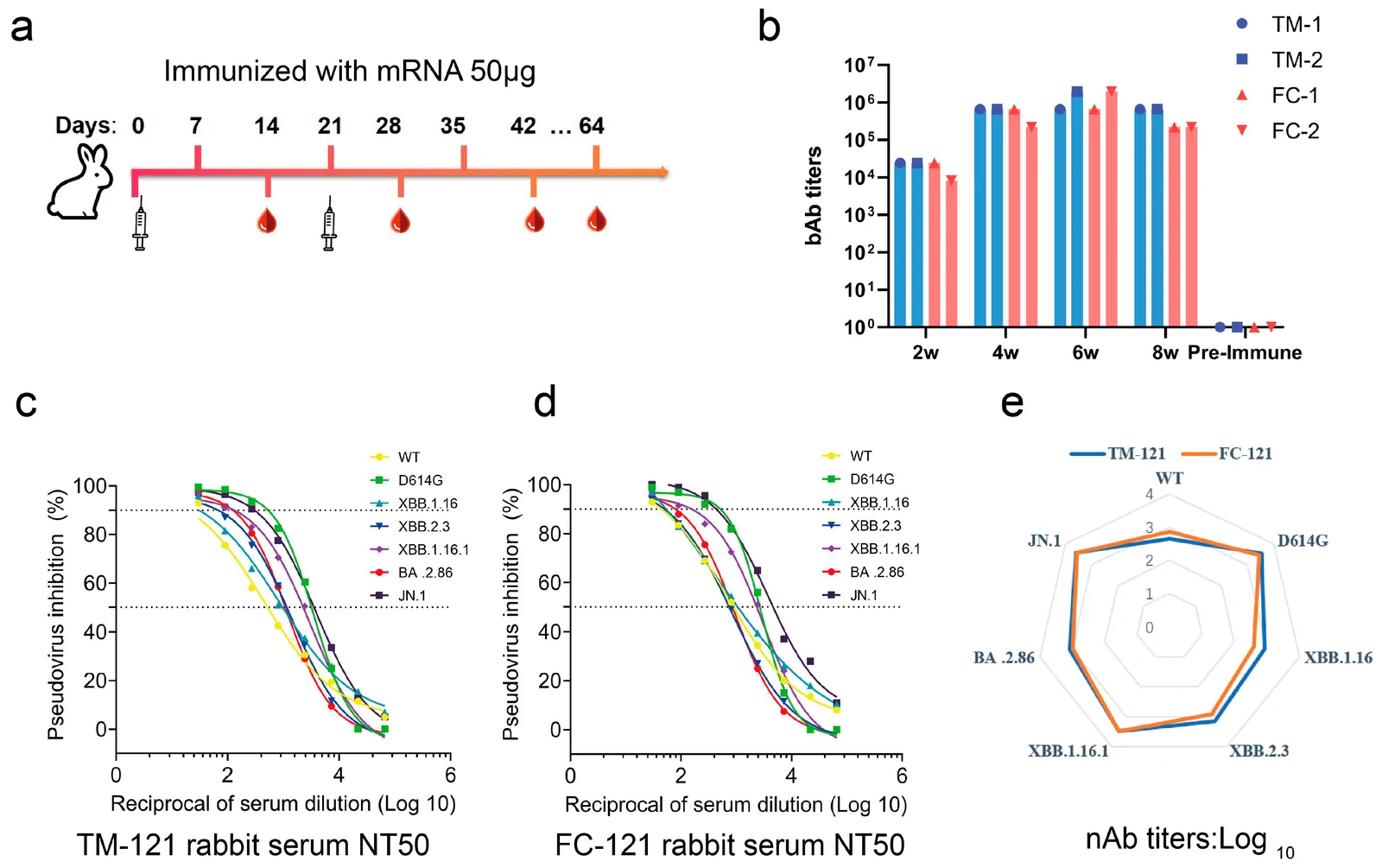
Robust and cross-variant neutralizing antibody responses in New Zealand White rabbits. (**a**) Timeline of the immunization regimen (t) and serum collection. (**b**) Serum IgG binding antibody titers against HR121 measured by ELISA. (**c**,**d**) Neutralization curves of sera from the (**c**) TM-HR121 and (**d**) Fc-HR121 groups against a panel of seven SARS-CoV-2 pseudotyped variants. (**e**) Radar chart illustrating the breadth of neutralization, with values representing the 50% neutralization titers (*NT*_50_). Data are presented as geometric mean ± geometric SD.

We subsequently evaluated the immunogenicity of the TM-HR121 and Fc-HR121 mRNA vaccine candidates in New Zealand White rabbits (Figure 2a). Following three immunizations with the two vaccines at three-week intervals, the rabbit sera were collected two weeks after the last immunization. Using enzyme-linked immunosorbent assay (ELISA), robust binding antibody titers in the sera were detected; the binding antibody (bAb) titers reached 1 × 10^6^ in both the TM-HR121 groups and the Fc-HR121 groups (Figure 2b). Then, the sera were evaluated for neutralizing activity against a comprehensive panel of pseudotyped SARS-CoV-2 variants, including WT, D614G, XBB.1.16, XBB.2.3, XBB.16.1, BA.2.86 and JN.1. In the TM-HR121 group (50 μg), the neutralizing antibody (nAb) titers (50% neutralization titers, NT_50_s) ranged from 459 against WT to 3859 against JN.1, with intermediate titers of 3541 (D614G), 851 (XBB.1.16), 1421 (XBB.2.3), 3050 (XBB.1.16.1), and 1182 (BA.2.86). In the Fc-HR121 group, the NT_50_ values ranged from 721 against WT to 3793 against JN.1, with intermediate titers of 2893 (D614G), 408 (XBB.1.16), 840 (XBB.2.3), 3031 (XBB.1.16.1), and 949 (BA.2.86). The sera in both groups exhibited potent neutralizing activity against all evaluated strains, with NT_50_s ranging consistently between 400 and 4000 (Figure 2c,d). Both mRNA vaccine candidates elicited broadly cross-reactive neutralizing antibody responses, as illustrated in a comprehensive radar chart analysis (Figure 2e). Both the TM-HR121 and Fc-HR121 mRNA vaccine candidates induced more robust and broader humoral immune responses in the rabbits.

### 3.3. TM-HR121 and Fc-HR121 mRNA Vaccines Elicited Superior Cellular and Humoral Immunity in Mice

**Figure 3:**
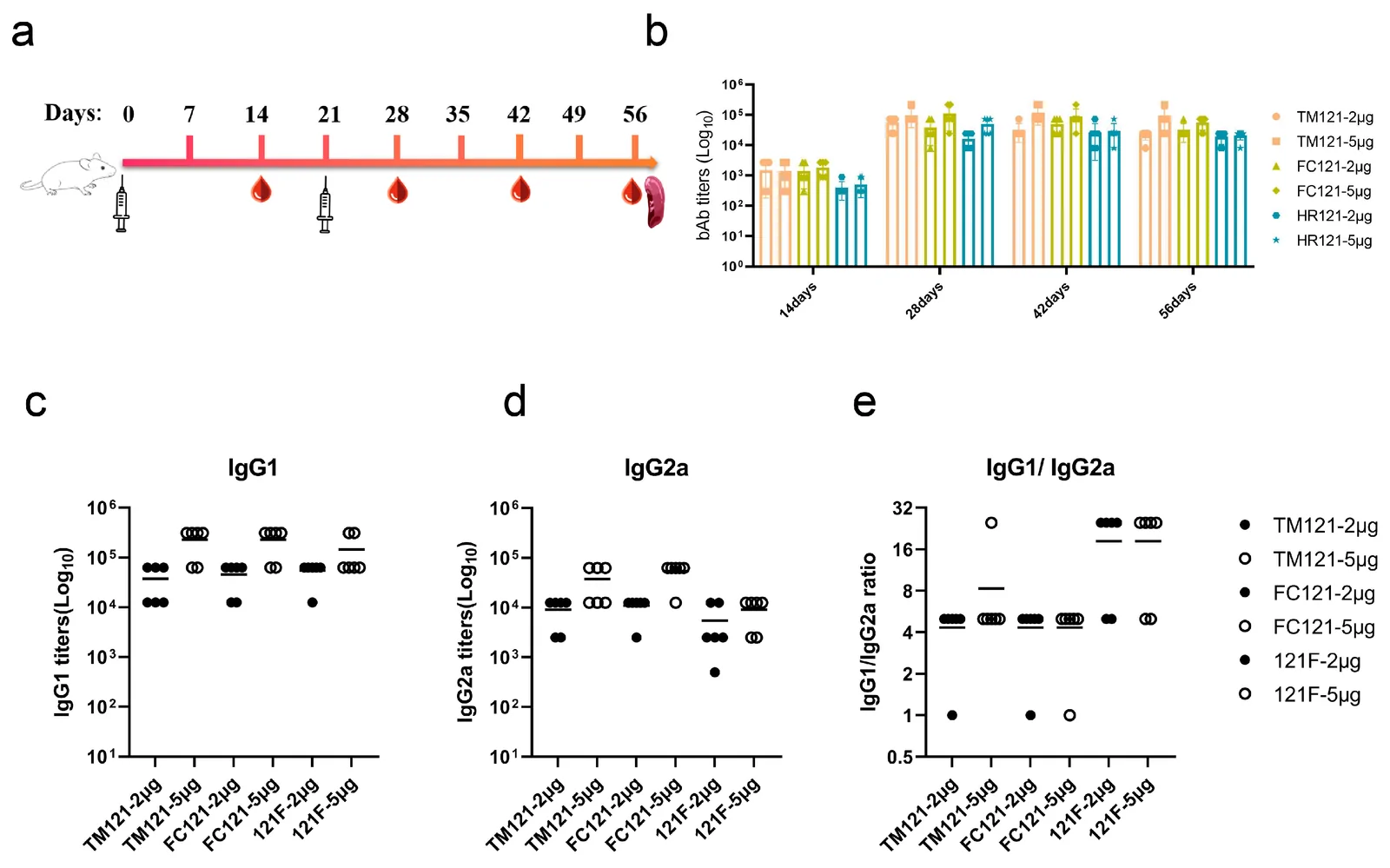
Balanced Th1/Th2 profiles in BALB/c mice. (**a**) Schematic of the murine immunization and splenocyte isolation protocol. (**b**) Total serum IgG titers measured by ELISA. (**c**) serum IgG 1 titers measured by ELISA. (**d**) serum IgG2a titers measured by ELISA. (**e**) Th1/Th2 skewing assessed by the ratio of IgG1 to IgG2a isotypes.

To further characterize the humoral and cellular immune responses elicited by TM-HR121 and Fc-HR121, we immunized BALB/c mice with the two vaccines using a regimen similar to that used in rabbits and assessed serum bAb titers via ELISA (Figure 3a). All vaccinated mice successfully showed high-level IgG antibody responses. HR121-specific IgG antibodies were induced in all vaccinated groups. The highest endpoint titers observed in the TM-HR121 and Fc-HR121 groups reached 32,400 and 48,600 at the 2 μg dose and increased to 121,500 and 89,100 at the 5 μg dose, respectively. In comparison, the HR121 protein vaccine group showed maximum endpoint titers of 27,000 and 29,700 at the corresponding doses. Overall, the mRNA-LNP vaccines achieved higher peak antibody responses, with approximately 1.20–4.09-fold increases compared with the protein vaccine control (Figure 3b). Analysis of antigen-specific IgG subclasses revealed that both mRNA vaccines elicited substantial IgG1 and IgG2a responses. The IgG1/IgG2a ratio in the mRNA-LNP groups was approximately 4, whereas the recombinant protein formulated with aluminum hydroxide adjuvant exhibited a ratio of approximately 15. Although the mRNA groups showed a higher proportion of IgG1, the IgG1/IgG2a ratio was significantly lower than that of the protein-alum group. In addition, the absolute IgG2a titers in the mRNA groups were higher than those in the protein-alum group, although the difference did not reach statistical significance. These data, together with the cytokine profiles described below, indicate that mRNA-LNP immunization induced a mixed Th1/Th2 response with a less pronounced Th2 bias than the alum-adjuvanted protein vaccine.

**Figure 4:**
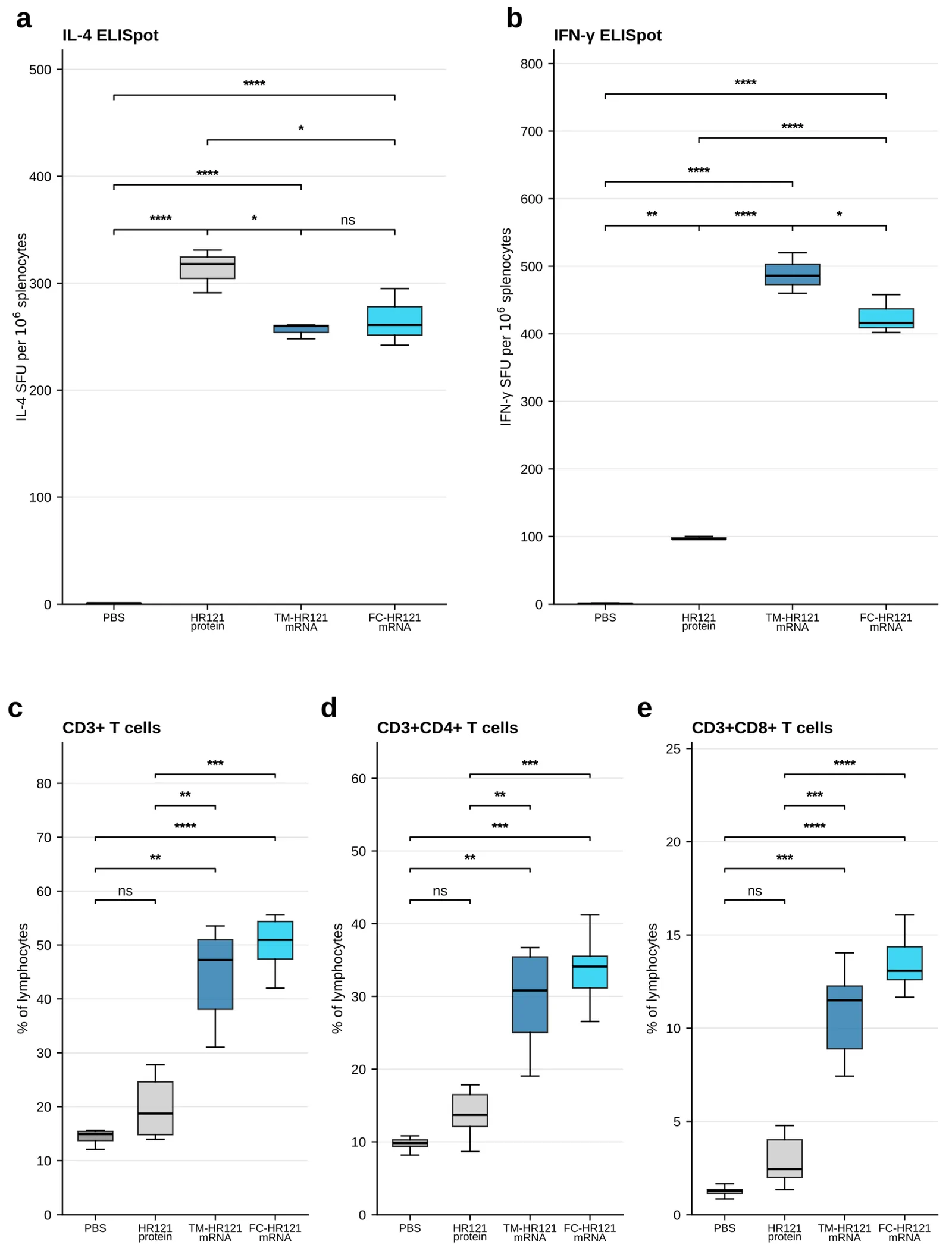
Cellular immune responses induced by HR121 vaccines in BALB/c mice. (**a**,**b**) Quantification of IL-4 and IFN-γ spot-forming cells by ELISpot. (**c**–**e**) Flow-cytometric quantification of splenic CD3^+^, CD3^+^ CD4^+^, and CD3^+^ CD8^+^ T cells. Boxplots show the median and interquartile range; whiskers extend to 1.5 × IQR. For panels (**a**,**b**), ordinary one-way ANOVA with Tukey’s multiple-comparisons test was used; for panels (**c**–**e**), Welch’s one-way ANOVA with Games-Howell post hoc comparisons was used. ns, not significant; * *p* < 0.05; ** *p* < 0.01; *** *p* < 0.001; **** *p* < 0.0001.

Subsequently, cellular immune responses evoked by the two mRNA vaccines were evaluated in splenocytes of the immunized mice using enzyme-linked immunospot (ELISpot) assays. Splenocytes isolated from immunized mice were stimulated ex vivo with HR121 peptide pools and analyzed by ELISpot assays. As expected, negligible cytokine secretion was detected in the PBS group. Compared with the HR121 protein vaccine, both TM-HR121 and Fc-HR121 elicited markedly stronger IFN-γ responses, with approximately 480 and 420 spot-forming cells (SFCs) per 10^6^ splenocytes, respectively, whereas the HR121 protein vaccine induced only approximately 90 SFCs (Figure 4a,b). In contrast, IL-4 secretion was readily detected following vaccination with all HR121-based formulations. The HR121 protein vaccine generated the highest IL-4 response (approximately 310 SFCs/10^6^ cells), while TM-HR121 and FC-HR121 induced moderate but comparable IL-4 responses (approximately 250 and 260 SFCs/10^6^ cells, respectively). These findings indicate that the mRNA vaccines preferentially enhanced antigen-specific Th1 cellular responses while maintaining detectable Th2 responses, resulting in a more balanced cellular immune profile than the recombinant protein vaccine. To further assess cellular immune responses, splenocytes were analyzed by flow cytometry. Both TM-HR121 and Fc-HR121 vaccination increased the frequencies of antigen-specific CD4^+^ and CD8^+^ T cells compared with the HR121 protein vaccine (Figure 4e; gating strategy shown in Figure S1a).

### 3.4. Protective Efficacy in a Golden Syrian Hamster Authentic Virus Challenge Model

**Figure 5:**
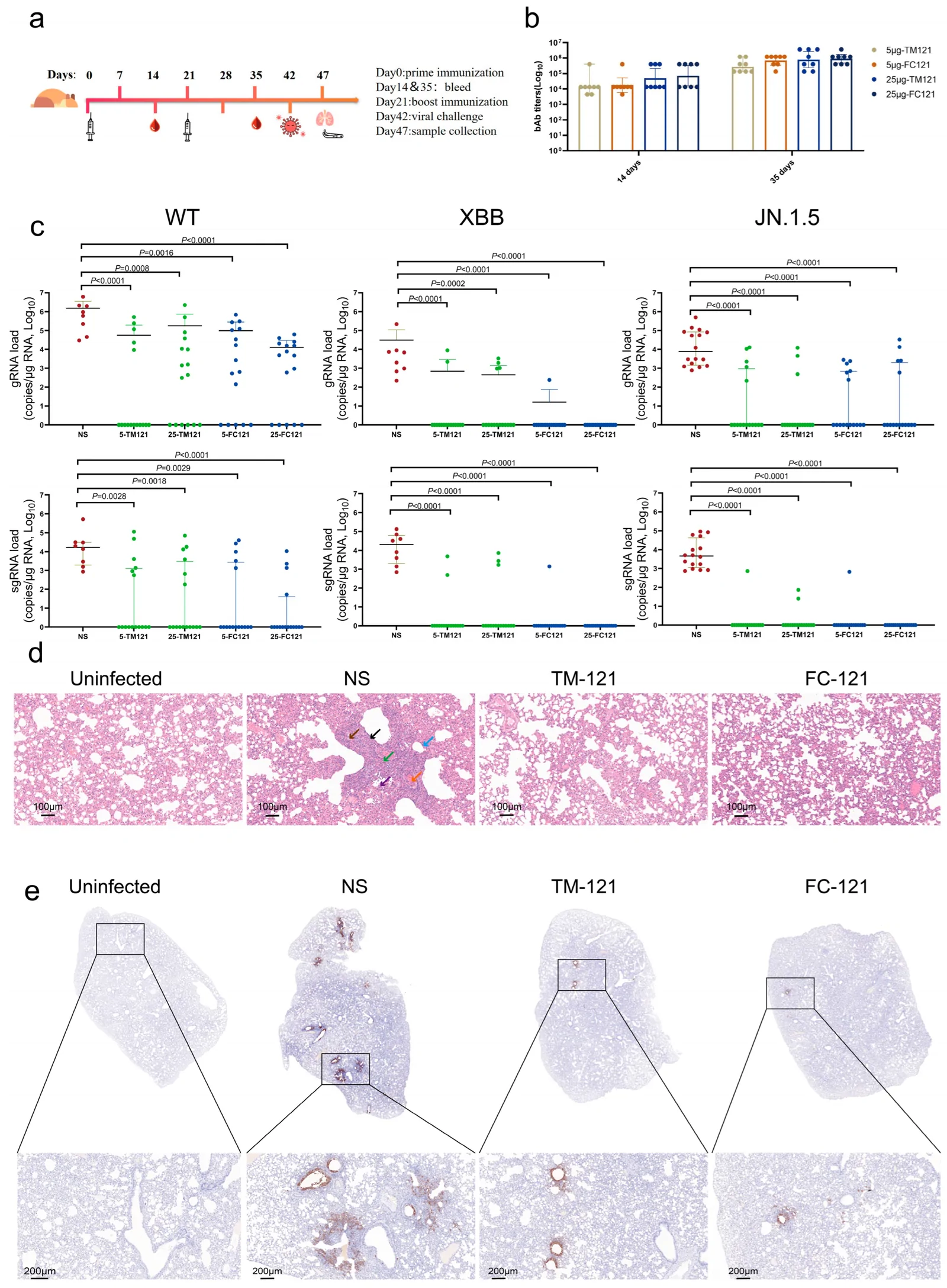
Protective efficacy of HR121 mRNA vaccines against authentic SARS-CoV-2 challenge in hamsters. (**a**) Experimental timeline for the immunization and authentic SARS-CoV-2 challenge in Golden Syrian hamsters. (**b**) Serum binding antibody titers prior to viral challenge. (**c**) Lung viral loads quantified by genomic RNA (gRNA) and subgenomic RNA (sgRNA) copies per gram of tissue at day 4 post-infection. (**d**) Representative histopathological H&E staining of lung sections; arrows indicate sites of inflammatory infiltration or bronchial damage (scale bars as indicated, red arrow: hemorrhage, black arrow: lymphocytes infiltration, blue arrow: alveolar shrinkage, and green arrow: alveolar collapse, purple arrow: inflammatory cell aggregation, brown arrow: positive viral antigen signal). (**e**) Immunohistochemical (IHC) staining of lung tissues for the SARS-CoV-2 nucleocapsid (N) protein. Data are expressed as median ± IQR.

To evaluate in vivo protective efficacy, we immunized Golden Syrian hamsters and subsequently challenged them with live SARS-CoV-2 VoCs (the prototype strain, XBB, and JN.1.5) (Figure 5a). Prior to challenge, all vaccinated cohorts had developed robust serum antibody responses (Figure 5b). At day 4 post-infection, lung viral loads were quantified by RT-qPCR. Whereas the normal-saline (NS) control animals carried high levels of viral genomic RNA (gRNA, ~8.1 log_10_ copies per gram) and subgenomic RNA (sgRNA, ~6.5 log_10_ copies per gram), immunization with TM-HR121 or Fc-HR121 reduced gRNA to ~3.1 and ~3.4 log_10_ copies per gram and sgRNA to ~1.4 and ~1.7 log_10_ copies per gram, respectively (Figure 5c). This corresponded to reductions in sgRNA of approximately 5.1 (TM) and 4.8 (Fc) log_10_ relative to the NS group, indicating effective suppression of viral replication by both mRNA vaccine candidates.

Consistent with the virological findings, hematoxylin-and-eosin (H&E) staining showed pronounced pulmonary damage in the NS cohort—including bronchial epithelial necrosis, inflammatory-cell infiltration and vascular congestion—whereas these lesions were attenuated in the vaccinated animals (Figure 5d). Blinded semi-quantitative scoring across all challenge groups (Figure S2) showed that the mean total lesion score was lower in every vaccinated group than in the corresponding NS control (for the prototype challenge, 5.75 in NS versus 4.6–5.1 across the vaccinated groups), although the magnitude of histological improvement was more modest than the reduction in viral load. Immunohistochemistry further supported this trend: residual viral nucleocapsid (N) protein, which was abundant in the lungs of the NS group, was markedly reduced to near-undetectable levels in the mRNA-vaccinated cohorts (Figure 5e). Taken together, the mRNA vaccines provided strong protection against challenge with multiple VoCs—most clearly at the level of viral replication and antigen burden—an effect that likely reflects their superior induction of T-cell-mediated immunity.

## 4. Discussion

Immune-evasive SARS-CoV-2 variants, characterized by accumulating mutations in the RBD and NTD of the S1 subunit, have significantly undermined the neutralizing efficacy of RBD-targeted vaccines [5,27]. Current mRNA vaccine strategies, which rely on RBD sequences, are increasingly limited by antigenic drift and need to be updated for the emerging VoCs [28]. Thus, it is essential to design vaccine immunogens focused on conserved, evolutionarily constrained epitopes.

Targeting conformational transitions of viral fusion proteins is an established strategy, as illustrated by prefusion-stabilized RSV F immunogens and the RSV-neutralizing monoclonal antibody nirsevimab [29,30]. Our previous work showed that HR121, a recombinant immunogen mimicking the SARS-CoV-2 S2 fusion intermediate, can induce broadly neutralizing antibodies [18]. Similarly, another recombinant protein, HR212, which targets the HR2 domain, also elicited broadly nAbs [14]. However, conventional recombinant protein immunogens are largely restricted to driving humoral antibody production and lack the capacity to potently activate cellular immunity, which renders their overall protective breadth inferior to that of mRNA vaccine platforms.

To address this limitation, we developed two mRNA-LNP vaccine candidates: TM-HR121, which incorporates a transmembrane domain to anchor the antigen on the cell surface, and Fc-HR121, which incorporates an Fc domain to promote dimerization. Both strategies were designed to stabilize the HR121 construct in a conformation that mimics the native S2 fusion intermediate, thereby exposing broadly neutralizing epitopes more efficiently than the recombinant protein vaccine.

In TM-HR121, the transmembrane anchor displays HR121 in a membrane-proximal conformation, recapitulating the natural topology of the S2 fusion machinery on virions and infected cells. This membrane-bound presentation may preserve conformational epitopes and enhance B-cell receptor engagement, as suggested by studies showing that membrane-anchored malaria transmission-blocking antigens induced stronger functional immune responses than their soluble counterparts [17]. Direct comparisons in clinical vaccine development have also favored membrane-bound, full-length spike over soluble RBD designs: BNT162b2, which contains the full-length S-2P with the TM domain, elicited broader spike-specific T-cell immunity and had a more favorable tolerability profile than BNT162b1, a secreted, soluble RBD without a TM domain [31]. Similarly, Moderna benchmarked mRNA-1273 against soluble or truncated spike constructs [32]. Although one study reported that deletion of the TM and cytoplasmic tail of SARS-CoV-2 spike did not impair neutralizing antibody induction [33], that analysis focused primarily on humoral responses and did not fully evaluate the breadth of T-cell epitopes or membrane-associated conformational epitopes, which are likely important for broad cross-variant protection.

The Fc-HR121 design intrinsically exploits the Fc domain to enhance antigen presentation, further amplifying this multifaceted humoral efficacy. In Fc-HR121, fusion to the human IgG Fc domain was designed to promote antigen multimerization, enhance molecular stability, and prolong serum half-life through FcRn-mediated recycling [34]. Fc fusion has been successfully employed in mRNA vaccines encoding RBD-Fc, which induced robust immune responses [35], and in a trivalent BVDV mRNA vaccine incorporating bovine IgG1 Fc to enhance antigen presentation [36]. Beyond stability, Fc fusion can facilitate antigen uptake by Fcγ receptors on antigen-presenting cells, potentially improving cross-presentation and T-cell priming. Consistent with this rationale, FcRn-guided antigen trafficking has been shown to enhance CD4^+^ and CD8^+^ T-cell responses in cancer vaccines [19].

Notably, TM-HR121 and Fc-HR121 induced similar humoral and cellular immune responses in mice and rabbits and conferred comparable protection in hamsters. This indicates that the HR121 antigen itself is the main driver of immunogenicity and that the addition of a transmembrane anchor or an Fc domain does not substantially alter the immune response under the immunization conditions used. Although Fc fusion can enhance antigen stability and uptake in some systems [19,35], these benefits may not be reflected in acute immunogenicity when the antigen is already efficiently expressed from mRNA [33]. Thus, the choice between TM and Fc fusion for this immunogen may be guided by manufacturing or pharmacokinetic considerations rather than by differences in immune activation.

Furthermore, unlike recombinant HR121 expressed in *E. coli*, which lacks critical post-translational modifications [13], the mRNA-encoded HR121 undergoes mammalian cell glycosylation. The S2 subunit is heavily shielded by a complex glycan network that governs antigen folding and immune recognition [9]; therefore, mammalian-cell glycosylation may contribute to the enhanced immunogenicity observed with the mRNA vaccines, although direct biochemical characterization of HR121-specific glycans has not yet been performed.

Beyond direct neutralization, antibody-dependent cellular cytotoxicity (ADCC) plays a pivotal role in SARS-CoV-2 clearance [18,24]. Unlike ADCC-mediating antibodies targeting the highly variable S1/RBD regions, which are readily evaded by emerging variants, antibodies specific to the evolutionarily conserved S2 domain may maintain stable Fc-dependent effector activities across divergent viral strains [37]. The conformational stability of HR121 ensures that the induced antibodies can durably recruit NK cells and macrophages to execute ADCC.

A major inherent limitation of conventional adjuvanted protein subunit vaccines is their tendency to skew toward a Th2-dominant humoral response, frequently failing to prime the potent CD8^+^ cytotoxic T cells required for eliminating virus-infected cells. In this study, the recombinant HR121 protein vaccine formulated with aluminum hydroxide exhibited an IgG1/IgG2a ratio of approximately 15, indicative of strong Th2 polarization. In contrast, the mRNA-LNP vaccines displayed an IgG1/IgG2a ratio of approximately 4, coupled with higher absolute IgG2a titers and robust IFN-γ-producing T-cell responses. We define “balanced” in this context as the induction of substantial levels of both IgG1 and IgG2a, with a ratio that does not reach the extreme Th2 skewing typically associated with alum-based formulations. A moderate predominance of IgG1, together with concurrent Th1-type cellular immunity, is considered favorable for antiviral protection, as it supports both antibody-mediated neutralization and cellular effector functions while reducing the risk of vaccine-associated enhanced respiratory disease [32,38]. Thus, although the mRNA vaccines did not achieve an IgG1/IgG2a ratio below 1, the overall cytokine and antibody subclass profile is consistent with a mixed Th1/Th2 response.

Overall, the findings generally support our hypothesis: both HR121 mRNA vaccines induced broader neutralizing activity, more balanced antibody isotype responses, and stronger antigen-specific cellular immunity than the recombinant HR121 protein and were associated with substantially reduced pulmonary viral RNA levels following authentic SARS-CoV-2 challenge. Nevertheless, the hypothesis was supported within the limits of the present small-animal study; pathological improvements were not uniformly significant, and the molecular contributions of antigen glycosylation or conformational differences were not directly established.

Despite the highly promising results in small animal models, we acknowledge several limitations in the present study. First, the protective efficacy of the HR121 mRNA vaccines has not yet been evaluated in non-human primate (NHP) models, a critical prerequisite for successful clinical translation. Second, although the immunogenicity and protective efficacy of the HR121 mRNA vaccine candidates were comprehensively evaluated, this study did not include a parallel comparison with a homologous recombinant HR121 protein vaccine under identical experimental conditions. Such a direct head-to-head comparison would provide a more rigorous assessment of the relative advantages of the mRNA platform and will be addressed in future studies. Third, given the rapid and continuous evolution of SARS-CoV-2, future investigations should assess the cross-protective durability of HR121 against updated VoCs (e.g., currently circulating JN.1-derived lineages such as NB.1.8.1, XFG, and BA.3.2), as well as the longevity of the induced immune memory. Nevertheless, current structural data suggest that the critical residues within the HR1/HR2 fusion machinery remain unaffected by recent mutations [39].

In summary, by combining the highly conserved HR1/HR2 structural domains with the advanced mRNA-LNP delivery platform, we have successfully developed two HR121-based mRNA vaccine candidates. This design strategy elicits a potent immune defense integrating both cross-variant humoral and cellular immunity, providing a robust strategy for the development of variant-proof SARS-CoV-2 vaccines and potentially pan-coronavirus vaccines.

## 5. Conclusions

TM-HR121 and Fc-HR121 mRNA vaccines, designed to target the highly conserved HR1 and HR2 domains of SARS-CoV-2, elicited enhanced humoral and cellular immune responses relative to recombinant protein immunogens. The two vaccines induced potent neutralizing antibodies against multiple SARS-CoV-2 variants and promoted significant T-cell expansion with elevated secretion of IFN-γ. Furthermore, mRNA vaccination significantly reduced viral replication in the lungs and mitigated severe histopathological lesions in Golden Syrian hamsters following challenge with the prototype strain, XBB, and JN.1.5 variants. These findings support the development of broadly protective vaccines against SARS-CoV-2 and related coronaviruses.

## Data Availability

The original contributions presented in this study are included in the article and Supplementary Materials; further inquiries can be directed to the first authors and corresponding authors.

## Supplementary Materials

The following supporting information can be downloaded at: https://www.mdpi.com/article/10.3390/vaccines14090831/s1, Figure S1: Representative gating strategy for splenic T-cell flow-cytometry analysis and representative cytokine ELISpot wells.; Figure S2. Extended lung histopathological scores after viral challenge.; Table S1: Sequence composition and structural domains of HR121-based vaccine constructs.; Table S2. SARS-CoV-2-S circulating variants constructed in this study.
