# Supplementary material for: mRNA Vaccines Targeting the Conserved SARS-CoV-2 Fusion Machinery Elicit Cross-Variant and Robust Immunity
Source: Vaccines (Basel). 2026 Sep 21;14(9):831. doi: 10.3390/vaccines14090831 (PMC13612069; doi:10.3390/vaccines14090831)
Supplement: Supplementary file 1 [file vaccines-14-00831-s001.zip › vaccines-4519361-supplementary.pdf]

Supplementary Materials:

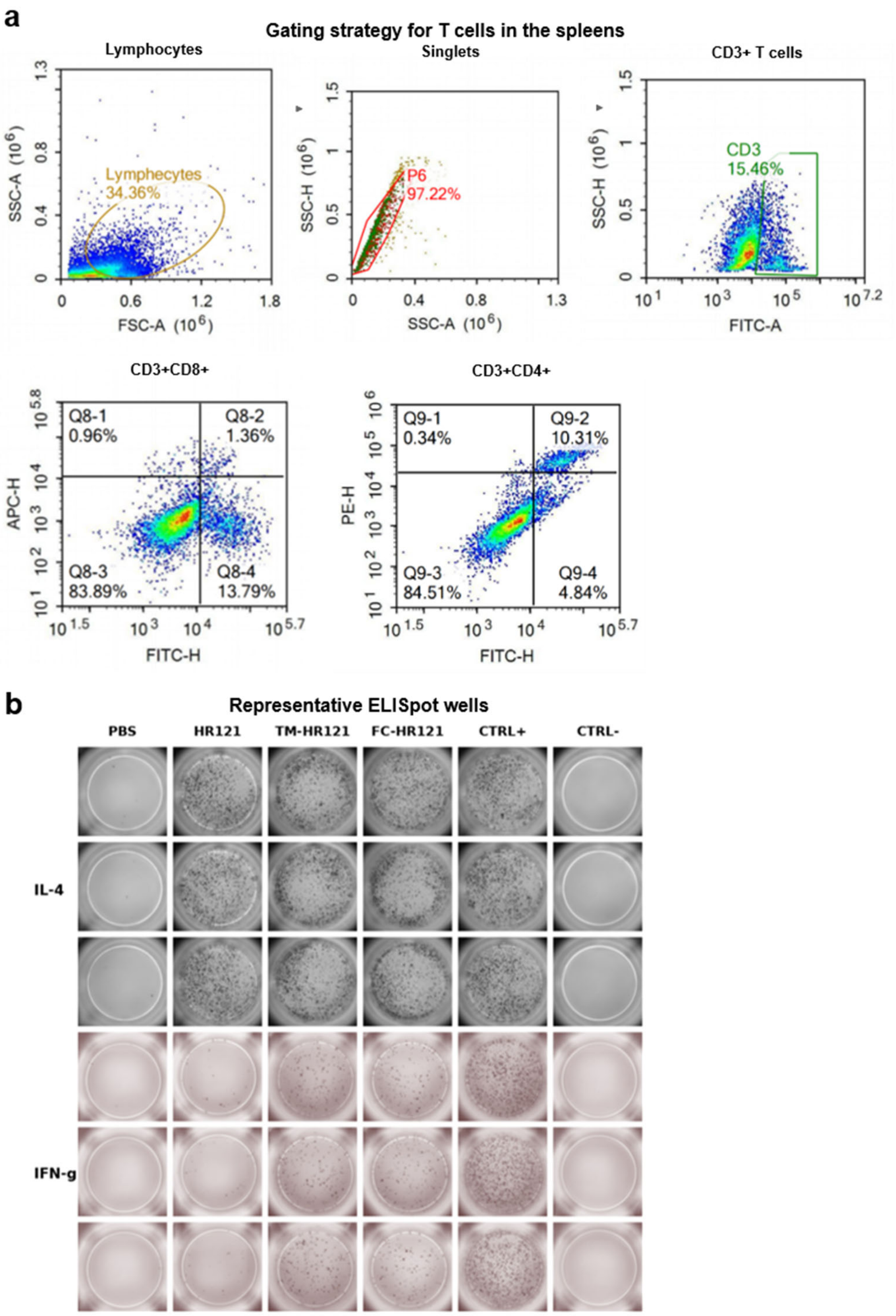

Figure S1. Representative gating strategy for splenic T-cell flow-cytometry analysis and representative cytokine ELISpot wells.

(a) Representative five-step gating strategy for splenic T-cell analysis from an Fc-HR121 mRNA-immunized mouse.

(b) Representative IL-4 and IFN-γ ELISpot wells from the PBS, HR121 protein, TM-HR121 mRNA, Fc-HR121 mRNA, positive-control, and negative-control groups. Quantification is shown in Figure 4.

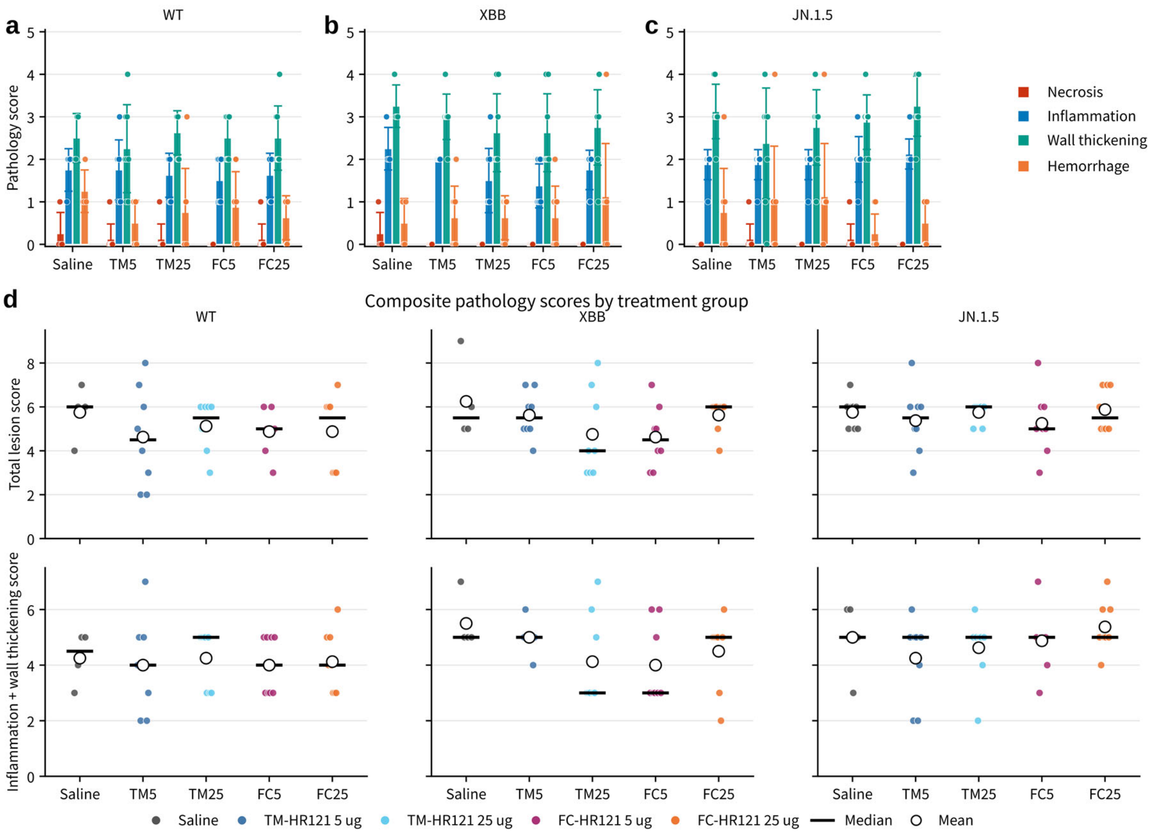

Figure S2. Extended lung histopathological scores after viral challenge. (a-c) Component pathology scores for WT, XBB, and JN.1.5 challenge groups, including necrosis, inflammatory-cell infiltration, alveolar-wall thickening, and hemorrhage. (d) Composite pathology scores by treatment group for the same challenge variants. Dots represent individual animals, horizontal bars indicate medians, and open circles indicate means. Treatment groups include saline control, 5 μ g and 25 μg TM-HR121, and 5 μg and 25 μg Fc-HR121. There is no significant difference between the vaccinated and control groups for all pathological parameters evaluated.

Table S1. Sequence composition and structural domains of HR121-based vaccine constructs.

name	sequences
TM-121	TQNVLYENQKLIANQFNSAIGKIQDSLSSTASALGKLQDVVNHNAQALN TLVKQLSSKFGAISSVLNDILSRLDKVEGGSGGDVDLGDISGINASVVNIQK EIDRLNEVAKNLNESLIDLQELGKYSGGRGGTQNVLYENQKLIANQFNSA IGKIQDSLSSTASALGKLQDVVNHNAQALNTLVKQLSSKFGAISSVLNDIL SRLDKVEEQYIKWPWYIWLGFIAGLIAIVMVTIMLCCMTSCCSCLKGCCSC GSCCKFDEDDSEPVLKGVKLHYT*
Fc-121	FDKTHTCPPCPAPELLGGPSVFLFPPKPKDTLMISRTPEVTCVVVDVSHED PEVKFNWYVDGVEVHNAKTKPREEQYNSTYRVVSVLTVLHQDWLNGKE YKCKVSNKALPAPIEKTISKAKGQPREPQVYTLPPSREEMTENQVSLTCLV KGFYPSDIAVEWESNGQPENNYKTTPPVLDSDGSFFLYSWLTVDKSRWQQ GNVFSCSVMHEALHNHYTQKSLSLSPGKGGGGSTQNVLYENQKLIANQF

	NSAIGKIQDSLSSTASALGKLQDVVNHNAQALNTLVKQLSSKFGAISSVLNDILSRLDKVEGGSGGDVDLGDISGINASVVNIQKEIDRLNEVAKNLNESLIDLQELGKYSGGRGGTQNVLYENQKLIANQFNSAIGKIQDSLSSTASALGKLQDVVNHNAQALNTLVKQLSSKFGAISSVLNDILSRLDKVEEQYIKWPWYIWLGFIAGLIAIVMVTIMLCCMTSCCSCLKGCCSCGSCCKFDEDDSEPVLKGVKLHYT*

* The linkers between HR1 and HR2; and Fc and HR1 are underlined.

Table S2. SARS-CoV-2-S circulating variants constructed in this study.

SARS-CoV-2-S circulating variants	Mutation sites
Omicron XBB.2.3	T19I, ΔL24, ΔP25, ΔP26, A27S, V83A, G142D, ΔY144, H146Q, E180V, Q183E, V213E, G252V, G339H, R346T, L368I, S371F, S373P, S375F, T376A, D405N, R408S, K417N, N440K, V445P, G446S, N460K, S477N, T478R, E484A, F486P, F490S, Q498R, N501Y, Y505H, P521S, T547I, D614G, H655Y, N679K, P681H, N764K, D796Y, Q954H, and N969K
Omicron BA.2.86	T19I, R21T, L24S, Δ25/27, S50L, Δ69/70, V127F, G142D, Δ144/144, F157S, R158G, N211I, Δ212/212, V213G, L216F, H245N, A264D, I332V, G339H, K356T, S371F, S373P, S375F, T376A, R403K, D405N, R408S, K417N, N440K, V445H, G446S, N450D, L452W, N460K, S477N, T478K, N481K, E484K, F486P, Q498R, N501Y, Y505H, E554K, A570V, D614G, P621S, H655Y, N679K, P681R, N764K, D796Y, S939F, Q954H, N969K, P1143L
Omicron JN.1	T19I, R21T, L24S, Δ25/27, S50L, Δ69/70, V127F, G142D, Δ144/144, F157S, R158G, N211I, Δ212/212, V213G, L216F,H245N, A264D, I332V, G339H, K356T, S371F, S373P, S375F, T376A, R403K, D405N, R408S, K417N, N440K, V445H, G446S, N450D, L452W, L455S, N460K, S477N, T478K, N481K, Δ483/483, E484K, F486P, Q498R, N501Y, Y505H, E554K, A570V, D614G, P621S, H655Y, N679K, P681R, N764K, D796Y, S939F, Q954H, N969K, P1143L
Omicron XBB.1.16	T19I, L24S, Δ25/27, V83A, G142D, Δ144/144, H146Q, E180V, Q183E, V213E, G252V, G339H, R346T, L368I, S371F, S373P, S375F, T376A, D405N, R408S, K417N, N440K, V445P, G446S, N460K, S477N, T478R, E484A, F486P, F490S, Q498R, N501Y, Y505H, D614G, H655Y, N679K, P681H, N764K, D796Y, Q954H, N969K
Omicron XBB.1.16.1	T19I, L24S, Δ25/27, V83A, G142D, Δ144/144, H146Q, E180V, Q183E, V213E, G252V, G339H, R346T, L368I, S371F, S373P, S375F, T376A, D405N, R408S, K417N, N440K, V445P, G446S, N460K, S477N, T478R, E484A, F486P, F490S, Q498R, N501Y, Y505H, T547I, D614G, H655Y, N679K, P681H, N764K, D796Y, Q954H, N969K
D614G	D614G
